# Size Matters: Zoo Data Analysis Shows that the White Blood Cell Ratio Differs between Large and Small Felids

**DOI:** 10.3390/ani10060940

**Published:** 2020-05-29

**Authors:** Sergey V. Naidenko, Mikhail V. Alshinetskiy

**Affiliations:** 1 Department of Behaviour and Behavioral Ecology, A.N. Severtsov Institute of Ecology and Evolution, Russian Academy of Sciences, Leninsky prospect 33, Moscow 119071, Russia; 2 Veterinary Department, Moscow Zoo, Bolshaya Gruzinskya str. 1, Moscow 123242, Russia; m.alshinetskiy@moscowzoo.ru

**Keywords:** felids, white blood cells, leukocytes, lymphocytes, neutrophils, monocytes, neutrophils/lymphocytes ratio, body mass

## Abstract

**Simple Summary:**

The white blood cells (WBCs) are some of the components of vertebrates’ immune systems. In some orders of mammals, the body mass of the animal correlates positively with the number of WBCs at the interspecific level. However, the different types of WBC play different roles in mammalian immunity, and we suggested that their number may vary between species as well. We estimated the number and ratio values of WBC types in 26 felid species and compared them with their body masses. We found that large cats had more neutrophils and monocytes and fewer lymphocytes than smaller ones. These differences may be explained by their diets. Large cats evolved as the hunters of medium and large-sized ungulates. They utilize the kills for long time intervals (days), resulting in the growth of fungi, protozoa and bacteria in the kills. That may explain the high number of neutrophils and monocytes in large cats to prevent infection by these organisms. The roles of different hunting styles, such as different times of kill utilization and the potential for a greater neutrophils/lymphocytes ratio in larger felids needs further investigation. This comparative study is helpful for zoo and wildlife veterinarians by allowing them to apply these results to endangered and poorly studied felid species.

**Abstract:**

The total number of white blood cells (WBCs) is related the immune system. In mammals, it is affected by the body mass, but it is unclear how the numbers of different WBC types correlate with this parameter. We analyzed the effect of body mass on WBC number and ratio in felids, where species are similar in diet (warm-blood vertebrates) and reproductive strategy (promiscuity). Based on zoo veterinary data (ZIMS database) we analyzed the effect of body mass on WBC number and neutrophils/lymphocytes ratio in 26 species of felids. The number of WBCs correlated with the body masses of animals: large cats had more WBC, which may be due to greater risks of infection associated with larger body surface, lifespan and home range size. For the first time we found obvious differences in the number of WBC types. Large cats also had more neutrophils and monocytes but fewer lymphocytes than smaller cats. The ratio of neutrophils to lymphocytes is greater in large felids. This phenomenon may be related to diet (relative prey size and kill utilization time), which suggests regular contact of large cats with bacterial and protozoal pathogens in contrast to the small cats.

## 1. Introduction

Hematological characteristics of different species depend on the habitats and environmental conditions where the species have evolved [1,2,3]. Usually, hematological analysis detects the parameters related to oxygen transportation (number of red blood cells (RBCs, erythrocytes), their volume, hemoglobin concentration and hematocrit), blood coagulation (platelets number and volume) and immunity (total number of white blood cells (WBCs, leukocytes) and their different types). Total number of WBCs and their different types as the index of immunity are the key points of several surveys in primates [4,5], carnivores [6] and rodents [7]. These multispecies studies determined some factors affecting the total number of WBCs in different species. Total WBC number in mammals correlated with species-specific body mass: larger species had more WBCs [5,6,7,8]. Whether WBC number and body mass are connected in carnivores remains largely unknown. Nunn and coauthors [6] has described the effects of different factors (body mass, mating strategy, life span, diet, habitats, age of sexual maturity, etc.) on WBC number in Carnivores. However, carnivores are a diversified taxon, which includes groups significantly differing in mean body mass (Ursidae are much bigger than Viverridae or Mustelidae) that also different in diet (Ursidae differ significantly from Felidae). It is easy to see that some of these parameters correlate with each other (body mass, life span and age of sexual maturity) or with the phylogeny of the species (for example, felids are the most highly specialized predators and have meat diets) [9]. Probably, phylogenetic status affects the results of the study (all felids are promiscuous [10,11] and Canids often form stable family groups [12,13]). The traits affecting the immune system (mating strategy, way of life, ratio of meat/plants in the diet, etc.) differ between the various groups of carnivores [6], and hence can bias the body mass effect. To exclude these factors, we need to analyze a less diversified taxon.

Felidae family is one of the most monomorphic among carnivores. It includes 41 species [14]. All felids demonstrate promiscuity as their main mating strategy [10]. Almost all wild felids are solitary (excluding lions [15] and cheetahs [16])) and terrestrial (excluding the arboreal clouded leopard [17]). Finally, the meat of warm-blooded vertebrates constitutes the main diet of almost all felids. Therefore, we have the opportunity to consider the relations of animals’ sizes (body mass) and WBC numbers in a monomorphic group of carnivores, similar in diet, lifestyle (terrestrial and solitary) and mating strategy (promiscuity). We hypothesized (1) that in felids larger species have higher total numbers of WBCs like in other taxa [4,5,6,7], and respectively, higher numbers of main WBC types (neutrophils and lymphocytes). However, different types of WBC have different functions: lymphocytes play an important part in forming antibodies and humoral immune response, but they are also involved in the cellular immune response [18]. Neutrophils are responsible for the phagocytosis of foreign cells [19] like monocytes that may serve to neutralize larger objects [20]. Eosinophils are involved in host defense against parasites and promoting allergic reactions, but also have series of regulatory functions [21]. The functions of basophils are still subject to discussion but they play an important role in interleukin production and are critically involved in a wide spectrum of immunologic disorders [22]. In theory (Hypothesis 2), an evolution in different ecosystems or phylogenetic relations of the species may affect the number of different WBC types depending on species-specific adaptation. This question was never studied in detail for carnivores, including felids. 

The aim of this study was to conduct a comparative analysis of total WBC number and the number of their different types and to correlate these data with the average body masses of different felid species. The understanding of the main trends in cellular immunity (number and ratio of leukocytes and their forms) will be useful for the different cat species in which hematological norms are absent and thus allow formulating new hypotheses for other groups of mammals. 

## 2. Materials and Methods

We used the international database ZIMS (2018, zims.species360.org) that accumulates the results of hematological measurements from the zoos over the world. We used the data on 26 felid species of 12 different genera (Table 1) (everything that was available in ZIMS at November of 2018, these materials are available as Supplementary Materials Table S1: Hematology ZIMS felidae Naidenko.). ZIMS data are presented as the mean of each index for each species. It also shows the number of tests and the number of tested individuals. We choose the species level and did not consider possible subspecies differences, although they were presented in ZIMS data base for some Felidae species. 

The database provided the average (mean) parameters for each species that we used for our analysis. These average indexes were calculated based on 15–3554 measurements (803 ± SE199) for 6–851 (209 ± SE49) individuals of each species (Table 1). Altogether, it included the data on 20,880 measurements of 5437 individuals. This is the largest data set, which is available for the analysis. The disadvantage of these data is that they do not include any raw data and do not give information about any animal’s diet, physiological status, age, sex, health status, lifestyle/animal management, stage of reproduction or entire/castrated status. That makes it more complicated to determine the differences between species (groups of species) because the data may be blurred. However, the large sample size (on average more than 200 animals and more than 800 samples) decreases or even neglects the effects of different factors (sex, age, etc.). 

We have analyzed the following parameters: total number of WBC; the number of their main types (lymphocytes, segmented and band neutrophils, monocytes, eosinophils, basophils) and their percentages; ratio of neutrophils–lymphocytes (calculated by ourselves as the number of neutrophils divided by the number of lymphocytes). We used two different approaches to estimate all parameters. The first one is the calculation of the correlation of the estimated parameters with the mean of species body mass of adult individuals. For the second approach all 26 species (the available data from ZIMS) were divided into large (body mass more than 30 kg, six species), medium (10–30 kg, eight species) and small (less than 10 kg, twelwe species) felids and all parameters were compared for these groups. To estimate the effect of body mass on WBC number we used the Kruskal–Wallis test. If the parameters from ZIMS database were counted using different methods (***A***, automated hemocytometer, manual (***M***, microscopy) or calculated (***C***, automatically measured WBC number by hemocytometer and recalculated based on smears microscopy), we used the one that described the maximal number of species. Finally, the A (automated) approach has been used for total WBC number and the ***C*** (calculated) method was applied for the number of all types of WBC. As such, we operate with one number (the mean) for each parameter (number of WBCs, neutrophils, monocytes, etc.) for each species, similar to earlier studies on primates [4,5], rodents [7] and carnivores [6]. 

As we mentioned above, we calculated the tests results based on species level and did not try to analyze subspecies level. In some cases, the taxonomy in ZIMS database was different to modern knowledge [14]; for example, only one species of clouded leopard (*Neofelis nebulosa* instead of two: *N. nebulosi* and *N. diardi*) was considered in ZIMS. In the present analysis, we were forced to follow these ZIMS data. Moreover, there were no data about sex and age of tested animals, and in theory, it may affect the results of this analysis (see Discussion). The mean body masses of adult individuals of felids were found in different references (Table 1). We attempted to not use the references for island subspecies or isolated populations. If the data were presented separately for males and females, we calculated average body masses for the adult animals.

## 3. Results

### 3.1. Larger Cats Have More WBCs Than Smaller Ones

**Figure 1:**
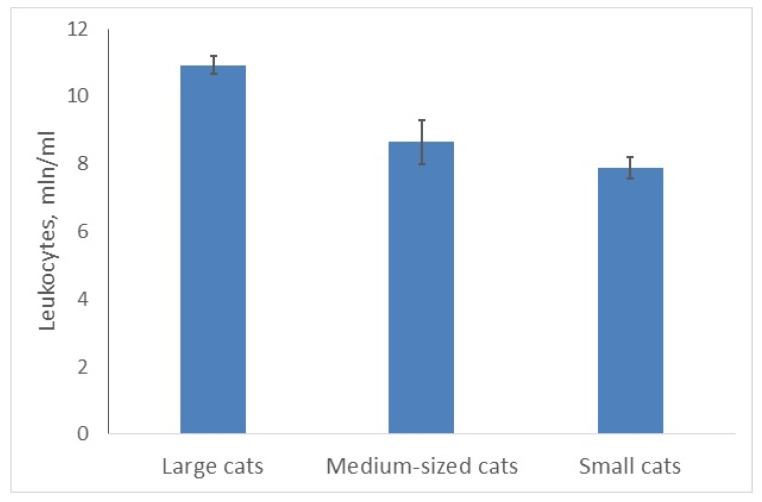
Average number of leukocytes in large felids is higher than in smaller ones.

The total number of WBCs varied significantly in felids of different sizes (***A,*** χ^2^ = 9.402; *p* = 0.0091; Figure 1). The minimal number of WBCs (7.88 ± 0.32 mln of cells per mL) was found in small cats; medium-sized cats had 10% more WBC; large ones—38% more. This number was maximal for the arboreal clouded leopard (medium-sized cat), the leopard and the lion (11.6–12.6 mln/mL). Another large felid (tiger; largest) had fewer WBCs (10.4 mln/mL). The world’s smallest wildcat (rusty-spotted cat) had the lowest number of WBC—5.9 mln/mL. At species level the mean number of leukocytes correlated positively with the mean body mass of adult animals (r = 0.53, n = 26; *p* < 0.05). 

### 3.2. Larger Cats Have More Neutrophils and Monocytes Than Smaller Felids

**Figure 2:**
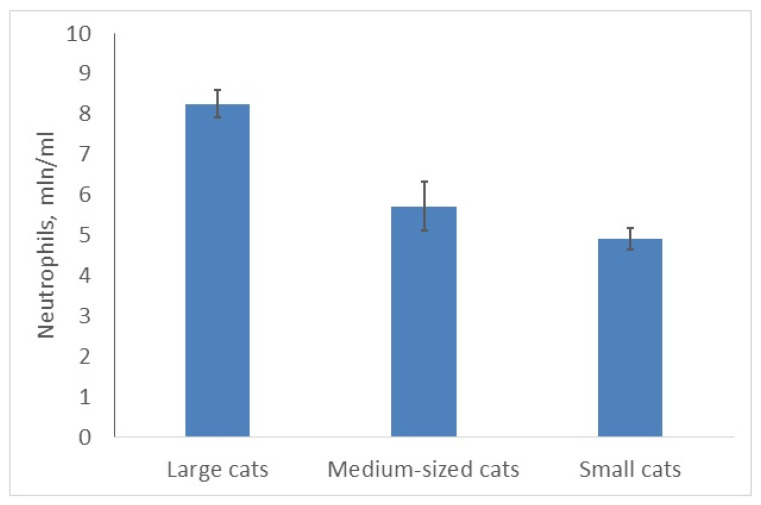
Larger felids have more neutrophils than smaller ones.

The situation is quite similar for the total number of neutrophils (as the main WBC type in cats) (Figure 2) and segmented neutrophils (as the main type of neutrophils). The size of animals affected both indexes significantly (***C,*** χ^2^ = 10.5; *p* = 0.0052 (22 species) and ***C,*** χ^2^ = 8.747; *p* = 0.0013 (25 species) respectively). Both indexes with the same coefficient (r = 0.6; *p* < 0.05) correlated with the body masses of the species. Leopards (large cat) and clouded leopards (medium-sized cat) had the highest numbers of neutrophils (9.9 mln/mL); the bobcat and the black-footed cat (both—small cats)—the lowest (3.8–4.4). Unfortunately, there were no data available on different leukocyte types in the rusty-spotted cat.

It is necessary to note that the mean numbers of eosinophils and basophils did not correlate with the sizes of cats; however, the number of monocytes (and neutrophils) was higher in large cats (***C,*** χ^2^ = 8.10; *p* = 0.034, 24 species) (r = 0.48; *p* < 0.05). Lions (large cat) and clouded leopards (medium-sized cat) had the highest numbers of monocytes (0.401–0.406 mln/mL); the bobcat and the Bengal cat (both small cats) had the lowest (0.176–0.213 mln/mL).

### 3.3. Small Felids Have More Lymphocytes and a Lower N/L Ratio Than Big Cats

**Figure 3:**
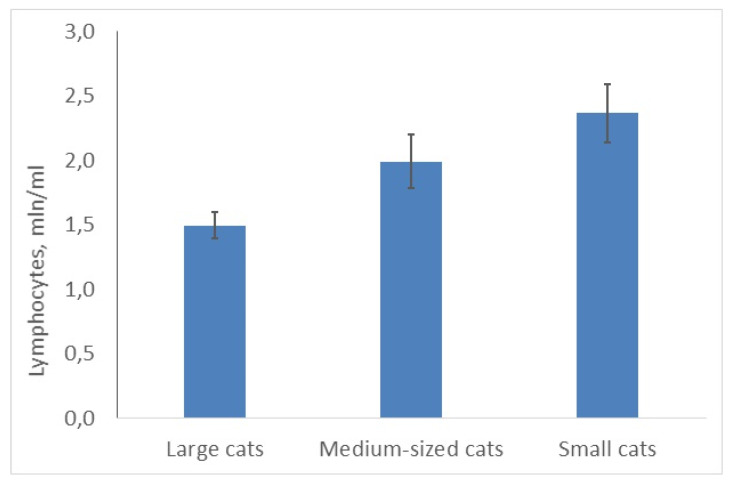
Average lymphocyte number is higher in smaller cats than in bigger ones.

However, the number of lymphocytes showed the opposite trend: it was significantly higher in small cats (***C,*** χ^2^ = 9.33; df = 2; *p* = 0.0094, 25 species) than in two other groups (Figure 3). The lymphocyte number correlated negatively and significantly with the mean body masses of species (r = −0.61; *p* < 0.05). Lymphocyte number was minimal in the snow leopard (large cat) (1.4 mln/mL) and maximal in the black-footed cat (small cat) (3.0 mln/mL). 

**Figure 4:**
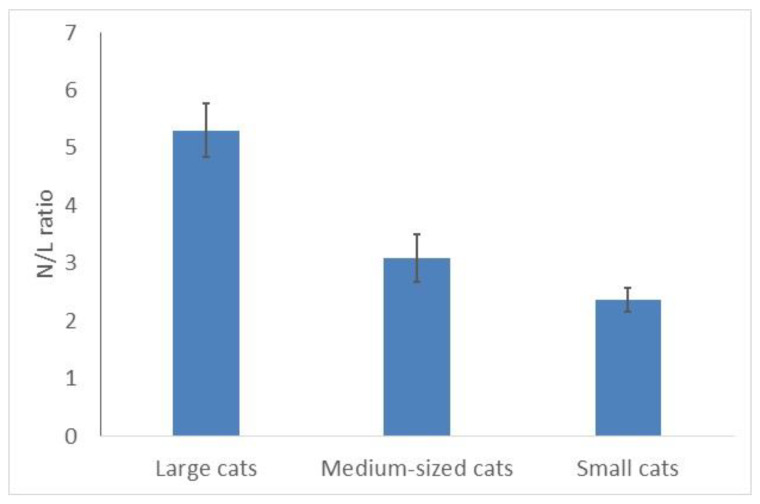
Mean neutrophils/lymphocytes ratio is higher in larger cats than in smaller felids.

Respectively, the ratio of neutrophils/lymphocytes (N/L) differed significantly in cats of different sizes (***C,*** χ^2^ = 9.33; *p* = 0.0094, 25 species) (Figure 4): it was the highest in large felids and the lowest in small cats (Figure 4). This ratio was two times higher in large cats (5.3) than in small ones (2.4). It correlated positively and significantly with the mean body masses of the species (r = 0.61; *p* < 0.05). Snow leopards and leopards (both are large cats) had the highest N/L ratios (6.3–6.6); Bengal and black-footed cats (both small cats)—the lowest (1.4–1.5). 

## 4. Discussion

WBC numbers in different mammalian orders varied significantly depending on the mean species-specific body mass [6,7]. Even in a monomorphic group such as felids, several differences in WBC number and ratio were detected. Larger cats (genera *Panthera* and *Acinonyx*) have a higher number (concentration) of WBCs, including neutrophils and monocytes, but a lower number of lymphocytes. Respectively, the ratio of neutrophils/lymphocytes was much higher in big cats. Medium-sized felids always had middle indexes (averaging between big and small cats). 

Potentially, the higher number of WBCs in bigger animals is an adaptation and may be related directly to the size of each animal. There are few hypotheses why larger animals may have high total WBC number as the first barrier of immune system. A larger animal implies a larger body surface, which may increase the risk of pathogens penetrating the host. Larger species may also have a higher infection risks than smaller species because larger bodies need more food and may harbor more pathogens than smaller ones [7,8]. In this case, bigger organisms are capable to produce more WBC. Larger felids move further daily and have bigger home ranges, which may increase the risk of contact with the pathogens [49,50]. Higher WBC concentration may provide the organism with a better defense in this case. 

The other hypothesis is related to the average lifespan of the species. As a rule, it is much shorter in small animals in comparison with the bigger ones. Longer lifespan may increase the probability of encountering pathogens, which makes keeping a higher leukocyte number in blood a valid adaptation. It is known, that in some carnivores with growing and maturation (and changes in food habits and lifestyle), the animals encounter pathogens more often [51]. Maintaining a high leukocyte number seems to be an adaptation to the higher number (over the whole life) of contacts with different pathogens for big, long-living animals. Regardless, independently of all these explanations, large cats have a higher number of WBCs than smaller ones, as we hypothesized. 

ZIMS database has some gaps, as it does not provide the information on individual animals (sex, age, reproductive status, etc.). Little is known about sex and age differences in WBC number and their main types’ ratio in mammals. The studies on humans show a clear effect of age on lymphocyte and neutrophil numbers (and their ratio) [52,53] and gender-related differences in post-surgery patients [54]. In laboratory animals (rats, mice and dogs), gender differences in WBC number were not detected [55,56]. Data for the model felid species (domestic cat) are very limited [57,58] (no gender-related differences) and nothing was published for the wild felids. Sex differences in hematological parameters may be not so important, but they may change intensively during pregnancy in females (in humans [59,60,61], cows [62], rats [63], dogs [64] and domestic cats [65]). In other studies, total and differential white blood cell counts have not been demonstrated to vary with pregnancy in domestic cats [66], but they vary in many other species [61] and pregnancy may be an important factor affecting the number of WBCs. Certainly, these factors (age, sex, reproductive status) may be important for the wild felids as well. The absence of these data in this study may, in theory, bias species-specific results in some cases. However, based on our collaboration with different zoos, blood sampling will be conducted much more often for adult (or subadult) animals than for younger ones. Normally, these sampling procedures are not biased to one sex and pregnant females are disturbed extremely rarely. This approach is common for all felids and the effects of these factors on differences of hematological parameters of small and large cats should be neglected. Some other factors may affect the number of WBCs or some of their types. Different kinds of diseases, especially causing inflammation, may change the numbers of WBC and neutrophils [67]. Changes in husbandry condition may affect WBC number; for example, shifting the neutrophils/lymphocytes ratio due the stress of individuals [68,69] or high population density [58]. The differences in studied parameters depending on sex, age, physiological status, etc., may affect the numbers of WBC in individual animals.

However, a large sample size for each species (see Table 1) minimizes the data bias. From another point of view, the differences in the number of tests and tested animals show that several samples come from the same individuals, but we cannot estimate how many. This fact obviously leads to some autocorrelation, which we cannot estimate based on ZIMS data. However, the high number of animals (on average—209 individuals for each species) used in this database dramatically raises the likelihood of the obtained results reflecting the differences at species level, but not on the individual level.

For the first time, we described significant interspecific differences in a number of different types of WBC. We assumed that an increase of WBC number in large felids implied that the number of all leukocyte types has increased respectively (Hypothesis 1). However, large felids have high numbers of neutrophils and monocytes, but the number of lymphocytes is much lower than in small cats. Because of the high concentration of neutrophils, the lymphocyte percentage decreased to 15% of the total leukocyte number in big cats. Thus, the ratio of neutrophils/lymphocytes is very high in large felids. The neutrophils/lymphocytes ratio is a good indicator of stress [68]; however, it is not affected by short-term immobilization and blood-sampling [69] and reflects mainly long-term stress [70]. The sampling procedures in zoos should not affect this parameter too intensively. Usually, the time lag till blood sampling less than one hour both for small and large cats. The neutrophils/lymphocytes ratio is stable over this time interval [69]. Husbandry conditions of cats determine their stress/welfare level in captivity [70,71], but there are no obvious reasons to suppose higher stress levels in larger cats, which may affect their N/L ratio. Oppositely, Amur tigers have a lower glucocorticoids concentration in captivity than in the wild [72]. In smaller cats, the presence of larger carnivores, changes of keepers and untypical enclosure interiors may lead to the stress in individuals (increase of glucocorticoids level) [70,71]. It seems that the stress factor cannot be the most relevant to the difference in the N/L ratio between large and small cats. Probably, the differences in WBC type ratios have been formed during the long-term evolutions of the species (Hypothesis 2).

The functions of different WBC types differ. Neutrophils play an important role in resistance to a variety of bacterial or fungal infections, killing penetrating pathogens by phagocytosis [19]. Monocytes have a similar function to neutrophils, but being macrophages, they are able to phagocytose pathogens of a larger size (mainly, bacteria, fungi and protozoa) [20]. Oppositely, lymphocytes play the key role in the development of humoral immune response, which provides resistance to viruses [18]. Could it be that smaller cats come in contact more often with the viral pathogens than larger cats—who may come in contact more with bacterial (fungal, protozoal) pathogens? These studies are absent. Geographical and biotopic variation in pathogens distribution make a comparative survey too complicated and low informative. In a case study which is conducted on a certain study site (it neglects the geographical effect) and uses few species, the researchers usually check the serum prevalence of 1–3 pathogens [73,74]. However, serum prevalence of different pathogens varies a lot and a small number of analyzed pathogens does not allow comparing small and large cats. The only example is a serum survey of four cat species, which was conducted in the Russian Far East for 15 different pathogens. Amur tigers and Far Eastern wildcats contacted seven and four (respectively) out of the nine tested viruses (no significant differences in number of pathogens). The percentages of tigers and wildcats serum-positive to the same viruses did not differ significantly for any of them [75]. However, different results have been obtained for the six tested non-viral pathogens [76]. Tigers had 3–6 times higher serum prevalence of *Trichinella* sp. and *Toxoplasma gondii* than wildcats (these differences were significant). Thus, tigers came into contact more often with some non-viral pathogens than wildcats [76]. 

Why in theory do large cats have greater contact with non-viral pathogens than smaller cats? These differences may be related to the diet specifics of these species, correlating with the functions of different WBC types in mammals. Felids mainly feed on warm-blooded animals; however, relative sizes of prey of large and small felids differ significantly. Normally, small felids prey on small animals (rodents, passerine birds or others)—their body masses being 10–100 times less than the predator’s body mass [9]. On the contrary, large felids prey mainly on different ungulates with body masses comparable with those of the predators (usually 80–88% and more) [77,78,79]. Large cats (excluding lion prides), are not able to eat the prey immediately and they forced to stay near the kill for a few days (sometimes for more than 1 week) [80,81]. In carnivores, species feeding on carrion are more likely to be infected than species feeding exclusively on freshly killed prey because of a higher abundance of pathogens colonizing carrion [82]. Moreover, felids live mainly in tropical/subtropical regions where even the short-term exposure of the kill to high air temperatures and high moisture results in intensive reproduction of bacterial and protozoal organisms (but not viral). This implies the regular contact of large cats with the non-viral pathogens on their kills. Small cats are able to eat the prey much faster (in a few minutes/hours) and they do not encounter this problem. That suggests higher exposure of large cats to non-viral pathogens than smaller cats and it may cause a higher level of neutrophils (and monocytes) and a higher neutrophils/lymphocytes ratio in large cats. It is interesting that the clouded leopard, which showed an extremely high numbers of WBCs and neutrophils, is a medium-sized cat, which preys on the species comparable with itself in body mass (monkeys, ungulates, etc.) and lives in humid hot tropical areas. Its specificity in WBC number may be explained by the similar diet to those of large felids. 

These findings also result in new hypotheses and raise new questions. For example, could these differences in numbers of WBC types be caused by other factors? Large and small cats separated from each other about 11 millions years ago [83], and do the neutrophils/lymphocytes ratios reflect the evolution of these two phylogenetic lines? If the differences in the neutrophils/lymphocytes ratio in felids are related to the diet traits, how will this hypothesis be supported by the data on other carnivores? For example, brown bears (*Ursus arctos*) and Asiatic black bears (*Ursus thibethanus*) are notorious for scavenging kills of tigers/leopards [84], but the sloth bear (*Melursus ursinus*) and sun bear (*Helarctos malayanus*) scavenge rarely, which will allow us to test this hypothesis on another group of carnivores. Most likely, the comparative survey studies of mammalian leukocytes will produce evidence to check this hypothesis.

## 5. Conclusions

In summary, the size of the felid correlates with the total leukocytes number and the neutrophils/lymphocytes ratio. It also affects the number of the main types of WBC: neutrophils, lymphocytes and monocytes. This point should be taken to account during the studies on rare felids in the wild and their management in captivity. Data on the studied species in zoos or closely related species should be used as references for the wildlife and zoo studies instead of the domestic cat data. 

## Figures and Tables

**Table 1:** Materials and Sources.

Species	Number of Tests *	Number of Animals *	Average Body Mass of Adult Individual	References for Body Mass
**Large cats**				
Tiger (*Panthera tigris*)	3485	848	170.0	[9]
Lion (*Panthera leo*)	2943	851	155.5	[23]
Jaguar (*Panthera onca*)	779	252	80.6	[24]
Leopard (*Panthera pardus*)	1008	283	50.0	[9,25]
Cheetah (*Acinonyx jubatus*)	3554	751	38.0	[26,27]
Snow leopard (*Panthera uncia*)	1642	438	35.0	[28,29]
**Medium sized cats**				
Mountain lion (*Puma concolor*)	1148	316	26.3	[30]
Eurasian lynx (*Lynx lynx*)	133	50	20.0	[31]
Clouded leopard (*Neofelis nebulosa*)	623	194	17.0	[32]
Serval (Leptailurus serval)	653	197	14.0	[32]
Fishing cat (Prionailurus viverrinus)	511	144	12.0	[33]
Caracal (Caracal caracal)	422	111	11.5	[34]
Asiatic golden cat (Catopuma temminckii)	73	31	10.7	[33]
Canadian lynx (*Lynx canadensis*)	313	115	10.5	[35,36]
**Small cats**	70			
Bobcat (*Lynx rufus*)	839	193	8.2	[37]
Ocelot (*Leopardus pardalis*)	758	153	7.8	[38]
Jungle cat (*Felis chaus*)	19	6	5.0	[39,40,41]
Bengal cat (*Prionailurus bengalensis*)	44	18	5.0	[40,41]
Jaguarundi (*Puma yagouaroundi*)	52	22	4.9	[38]
European wildcat (*Felis silvestris*)	238	40	4.5	[42]
Geoffroy’s cat (*Leopardus geoffroyi*)	47	17	4.3	[43]
Margay (*Leopardus wiedi*)	109	32	3.9	[44]
Pallas’ cat (*Otocolobus manul*)	581	157	3.7	[45]
Black-footed cat (*Felis nigripes*)	499	106	1.6	[46,47]
Sand cat (*Felis margarita*)	392	105	1.5	[9]
Rusty-spotted cat (*Prionailurus rubiginosus*)	15	7	1.3	[48]

* Both parameters varied depending on the test. Here we show the n for the automated white blood cells count.

## Supplementary Materials

The following are available online at https://www.mdpi.com/2076-2615/10/6/940/s1, Table S1: Hematology ZIMS felidae Naidenko.
